# Multidisciplinary Surgical Management of Radiation-Induced Colorectal Stricture and Pelvic Adhesions: A Case Report

**DOI:** 10.7759/cureus.103649

**Published:** 2026-02-15

**Authors:** Talha A Khan, Jay Swami

**Affiliations:** 1 Medical School, Cleveland Clinic Florida, Weston, USA; 2 Surgery, Cleveland Clinic Florida, Weston, USA

**Keywords:** colorectal stricture, high-output ileostomy, ileoureteral conduit, multidisciplinary surgery, pelvic adhesions, radiation proctitis

## Abstract

Radiation-induced colorectal strictures represent a challenging late complication of pelvic malignancy treatment, often accompanied by extensive fibrosis and multi-organ involvement. We report the case of a 55-year-old female with a history of cervical cancer treated with pelvic radiation, complicated by radiation proctitis, diverticular disease, pelvic adhesions, and bilateral ureteral obstruction, who underwent a single-stage multidisciplinary surgical reconstruction. The procedure included colostomy takedown, rectosigmoid resection with coloanal anastomosis, pelvic abscess debridement, diverting loop ileostomy creation, total abdominal hysterectomy, ileoureteral conduit formation, and ventral hernia repair with myocutaneous flap closure. The postoperative course was complicated by a high-output ileostomy resulting in electrolyte derangements, acute kidney injury, and malnutrition, requiring coordinated medical management with antidiarrheal therapy, electrolyte repletion, and nutritional support. This case underscores the complexity of late radiation sequelae and highlights the importance of multidisciplinary collaboration in achieving successful surgical and medical outcomes.

## Introduction

Radiation-induced colorectal strictures and complex pelvic adhesions are complications of pelvic radiation therapy, associated with impaired bowel function and significant morbidity [1,2]. Radiation proctitis, characterized by progressive fibrosis, ischemia, and mucosal injury, represents a common etiologic factor leading to stricture formation [2]. In patients treated for cervical cancer, pelvic radiation may also result in urologic sequelae, including bilateral ureteral obstruction, further compounding surgical complexity and management [3].

Surgical intervention in this setting presents a multidisciplinary challenge, necessitating a coordinated approach that integrates colorectal, urologic, gynecologic, and reconstructive techniques. Procedures such as colostomy takedown, rectosigmoid resection with coloanal anastomosis, pelvic abscess debridement, diverting loop ileostomy creation, total abdominal hysterectomy, ileoureteral conduit formation, and ventral hernia repair with myocutaneous flap closure are associated with increased incidence of postoperative complications [4].

A frequent postoperative complication in these patients is high-output ileostomy, which may precipitate dehydration, electrolyte derangements, malnutrition, and acute kidney injury [5]. High-output ileostomy is defined as an output of >1.5-2.0 L per 24 hours, though it varies according to the amount of oral intake. Effective management requires close monitoring, fluid and electrolyte replacement, and targeted nutritional support to mitigate morbidity [6,7]. Radiation-induced colorectal strictures develop in approximately 5-15% of patients following pelvic radiation therapy, with chronic radiation proctitis occurring in up to 20% depending on radiation dose and duration of follow-up [2,8]. Urologic complications, including ureteral obstruction and fistulization, have been reported in 2-10% of patients treated with pelvic irradiation for gynecologic malignancies [3,9]. However, the need for simultaneous colorectal, urologic, and gynecologic reconstruction remains uncommon and is typically reserved for advanced cases. Most reported surgical approaches favor staged reconstruction due to the elevated risk of anastomotic failure and postoperative morbidity [4,10]. Reported complication rates in these combined procedures exceed 30-50%, particularly in patients with prior radiation exposure [4,11].

In this report, we describe the case of a 55-year-old female with radiation-induced colorectal stricture, extensive pelvic adhesions, prior cervical cancer, and bilateral ureteral obstruction who underwent a complex single-stage surgical reconstruction. We highlight the operative considerations and postoperative medical management, particularly the treatment of high-output ileostomy, to illustrate the necessity of multiple departments working cohesively.

## Case presentation

A 55-year-old female with a surgical history of radiation proctitis secondary to prior pelvic radiation for cervical cancer, diverticulitis, prior colostomy, ventral hernia, pelvic adhesions, and bilateral ureteral obstruction presented for an elective operation due to a progressive radiation-induced colorectal stricture. These sequelae reflected advanced late-stage radiation injury involving multiple pelvic organ systems. She additionally exhibited mild malnutrition and intermittent electrolyte disturbances, raising concern for limited physiologic reserve.

**Figure 1:**
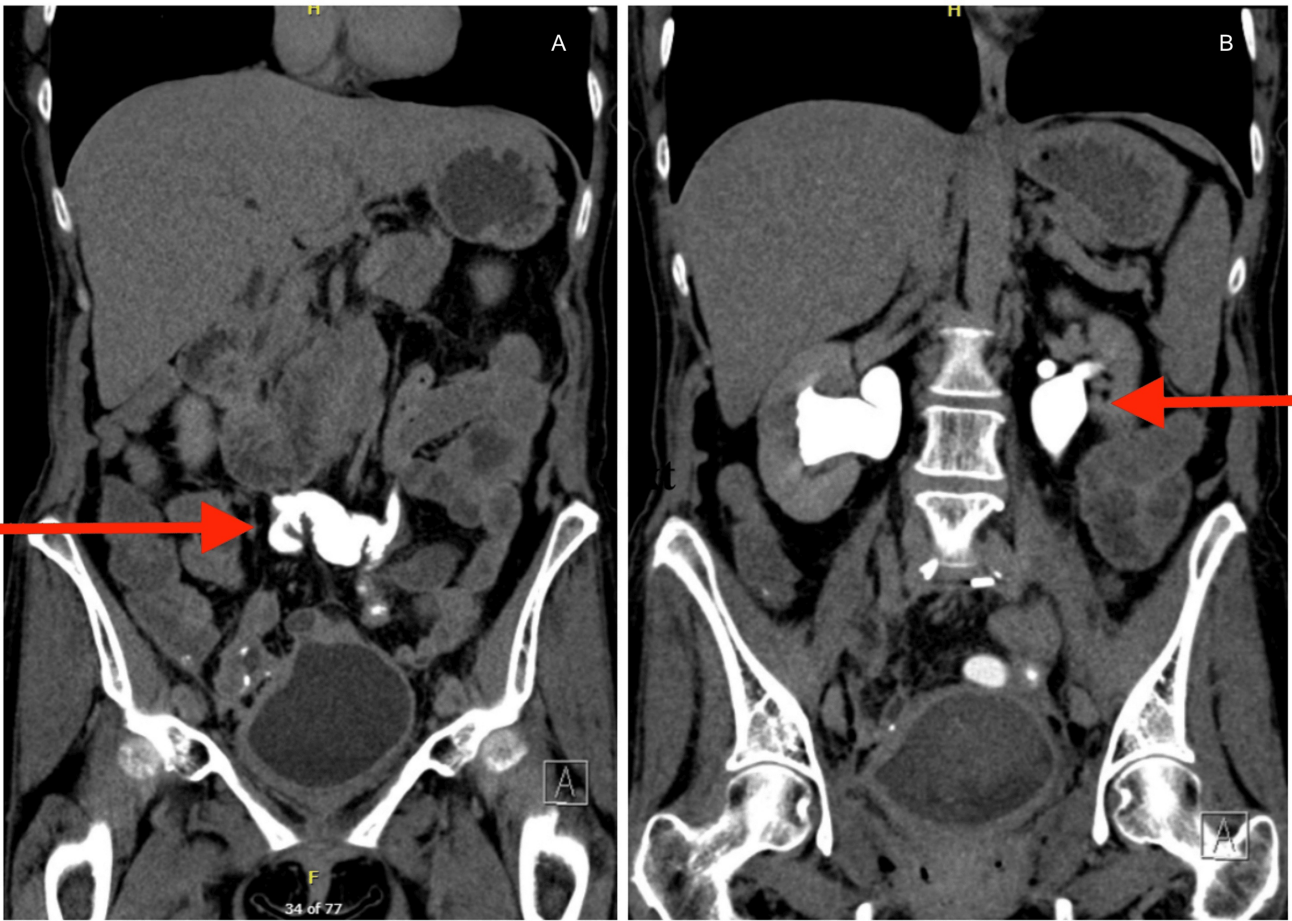
CT urogram a: coronal CT image demonstrating the urinary drainage site (red arrow) following distal ureteral resection with creation of a single ileal conduit connecting the bilateral ureters to the bladder dome; b: coronal CT image demonstrating bilateral hydroureteronephrosis (red arrow) status post double-J nephroureteral stent removal, likely secondary to vesicoureteral reflux. Postsurgical changes include prior abdominopelvic resection with left lower quadrant ileostomy and coloanal anastomosis.

On preoperative evaluation, the patient was hemodynamically stable and in no acute distress. Laboratory studies demonstrated mild hyponatremia and elevated blood urea nitrogen (BUN) (42 mg/dL) with preserved renal function (creatinine 0.78 mg/dL), findings that were suggestive of pre-renal azotemia in the setting of suspected volume depletion. Liver enzymes were elevated with values of alanine aminotransferase (ALT): 154, aspartate aminotransferase (AST): 101, and alkaline phosphatase (ALP): 661 (Table 1). CT imaging at this time demonstrated bilateral hydroureteronephrosis; postoperative changes included abdominopelvic resection with a left lower quadrant ileostomy and coloanal anastomosis (Figure 1). Given the extent of the disease and prior interventions, surgical planning required a coordinated multidisciplinary approach. The colorectal department serves as the primary team for the takedown of the existing ileostomy, extensive adhesiolysis, resection of diseased rectosigmoid segments, and reconstruction via coloanal anastomosis. Urology was consulted for bilateral hydroureteronephrosis and a history of ureteral involvement, and gynecology provided operative plans given the patient’s prior gynecologic malignancy and extensive pelvic adhesions involving reproductive structures.

**Table 1:** Pre-op labs

Laboratory Test	Patient Value	Normal Range
Sodium (Na⁺)	128 mmol/L	135–145 mmol/L
Alanine Aminotransferase (ALT)	154 U/L	5–40 U/L
Aspartate Aminotransferase (AST)	101 U/L	5–40 U/L
Alkaline Phosphatase (ALP)	661 U/L	150–420 U/L
Blood Urea Nitrogen (BUN)	42 mg/dL	5–18 mg/dL
Creatinine	0.78 mg/dL	0.3–0.7 mg/dL

The patient underwent a single-stage operative intervention consisting of colostomy takedown, exploratory laparotomy with extensive lysis of adhesions, rectosigmoid resection with coloanal anastomosis, pelvic abscess debridement, creation of a diverting loop ileostomy, total abdominal hysterectomy, ileoureteral conduit formation for urinary diversion, and ventral hernia repair with myocutaneous flap closure. Owing to the complexity of the procedure and anticipated postoperative risks, she was admitted to the intensive care unit for close monitoring.

**Figure 2:**
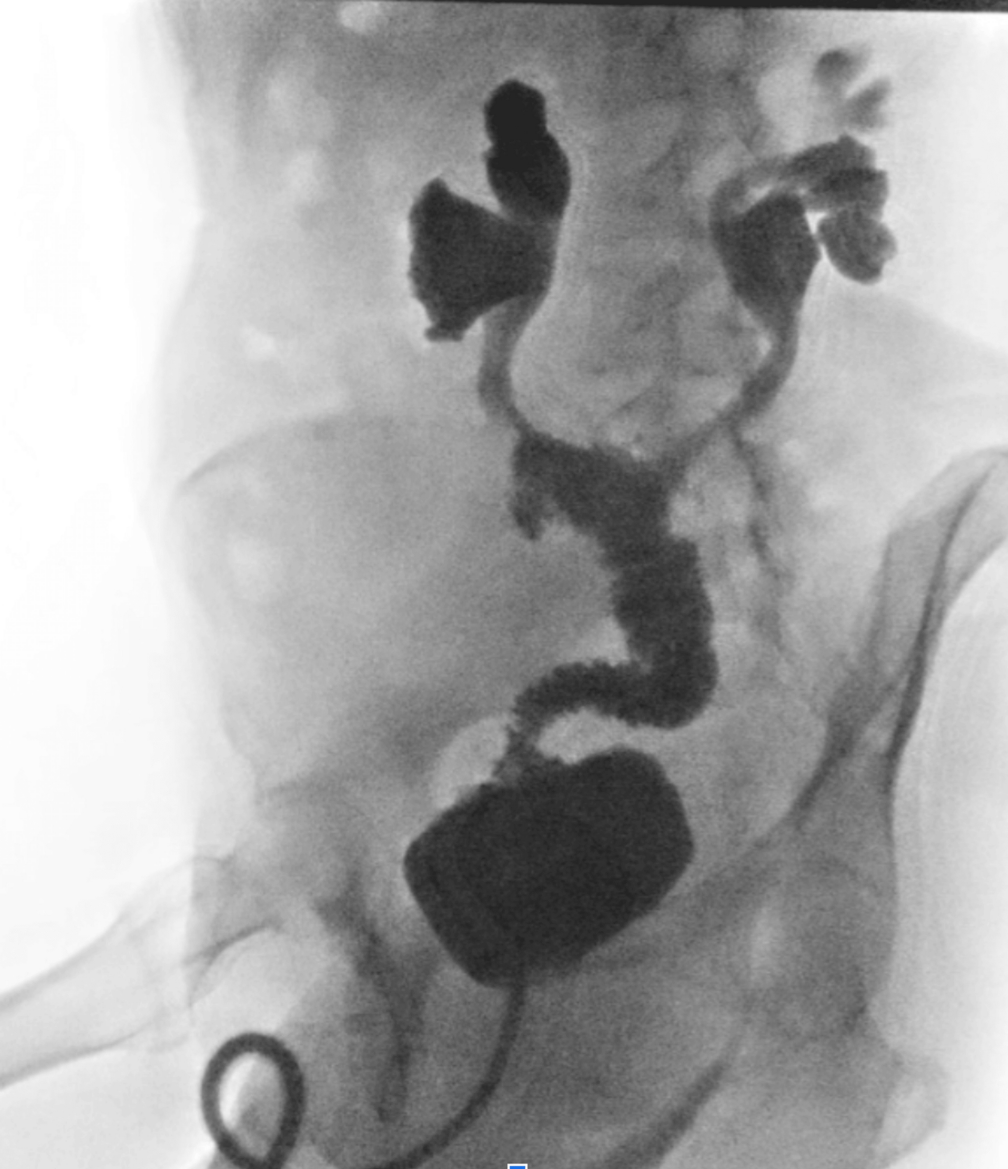
Post-procedural X-ray cystogram Demonstrating no evidence of leak/obstruction status post distal ureterectomy with interposed ileo-bladder anastomosis. Free contrast reflux to the kidneys with bilateral hydronephrosis.

On postoperative day one, the patient was successfully extubated and transferred to the surgical floor. Her postoperative course was complicated by persistently high output from the newly created ileostomy, resulting in hypokalemia and hypophosphatemia. These abnormalities were managed with scheduled antidiarrheal therapy, including loperamide and diphenoxylate-atropine, in addition to aggressive oral and intravenous electrolyte repletion. Renal function was closely monitored due to the combined risks posed by high ileostomy output and urinary diversion, though creatinine levels remained stable throughout hospitalization. The patient tolerated advancement to a gastrointestinal soft diet, and pain was controlled with a combination of intravenous and oral analgesics. A Foley catheter was maintained with plans for outpatient cystographic evaluation prior to removal (Figure 2).

She was discharged home with multidisciplinary outpatient follow-up, including home health services for wound care, electrolyte surveillance, and nutritional optimization.

## Discussion

This case highlights the challenges of managing a patient with complex radiation-induced pelvic disease. Radiation proctitis and fibrosis frequently lead to anastomotic strictures, which increase the risk of tissue friability, poor healing, and postoperative infection [1,2]. In this patient, bilateral ureteral obstruction necessitated the creation of an ileoureteral conduit, creating further surgical complexity.

The most significant postoperative concern was a high-output ileostomy, commonly defined as stoma output exceeding 1.5-2.0 liters per day, which is frequently observed following low pelvic anastomosis and small bowel diversion. Excessive stoma output can precipitate profound fluid and electrolyte imbalances. In this case, the patient developed multiple derangements, including pre-renal azotemia, as evidenced by elevated BUN with preserved creatinine, which required aggressive fluid and electrolyte management in the ICU [5,6].

Nutritional challenges were also prominent. The patient had baseline malnutrition, postoperative catabolism, and limited enteral tolerance due to high ileostomy output. Total parenteral nutrition was administered perioperatively, which may be associated with transaminitis and cholestasis and could have contributed to the observed elevation in liver enzymes and ALP [4].

Additional surgical considerations included abdominal wall reconstruction with a myocutaneous flap, which carries risks of flap failure and seroma formation [8]. Close monitoring, ICU support, and careful pain management facilitated recovery.

## Conclusions

This case highlights important clinical aspects of management: radiation-induced pelvic fibrosis complicates surgical intervention and healing, while high-output ileostomy requires prompt fluid, electrolyte, and nutritional management. Ileoureteral conduit creation represents a viable option for bilateral ureteral obstruction in irradiated fields when reconstructive alternatives are limited, while myocutaneous flap reconstruction, though effective, adds postoperative complexity requiring coordinated multidisciplinary care: colorectal, urologic, and reconstructive teams. Postoperative challenges, including high-output ileostomy, electrolyte disturbances, malnutrition, and risks associated with flap closure, were addressed through vigilant ICU monitoring, pharmacologic support, and nutritional optimization. This case underscores the value of integrated, multidisciplinary strategies in achieving favorable outcomes in patients with complex radiation-related pelvic disease.

## References

[1] Ashburn JH, Kalady MF (2016). Radiation-induced problems in colorectal surgery. Clin Colon Rectal Surg.

[2] McKeown DG, Goldstein S, Gasalberti DP (2025). Radiation proctitis. StatPearls [Internet].

[3] Feng MI, Bellman GC, Shapiro CE (1999). Management of ureteral obstruction secondary to pelvic malignancies. J Endourol.

[4] Podda M, Sylla P, Baiocchi G (2021). Multidisciplinary management of elderly patients with rectal cancer: recommendations from the SICG (Italian Society of Geriatric Surgery), SIFIPAC (Italian Society of Surgical Pathophysiology), SICE (Italian Society of Endoscopic Surgery and new technologies), and the WSES (World Society of Emergency Surgery) International Consensus Project. World J Emerg Surg.

[5] Rowe KM, Schiller LR (2020). Ileostomy diarrhea: pathophysiology and management. Proc (Bayl Univ Med Cent).

[6] Krishnamurty DM, Blatnik J, Mutch M (2017). Stoma complications. Clin Colon Rectal Surg.

[7] Kehlet H, Wilmore DW (2002). Multimodal strategies to improve surgical outcome. Am J Surg.

[8] Mates SJ, Steinwald PM, Foster RD, Hoffman WY, Anthony JP (2000). Complex abdominal wall reconstruction: a comparison of flap and mesh closure. Annals of Surgery.

[9] Andreyev HJ, Wotherspoon A, Denham JW, Hauer-Jensen M (2011). "Pelvic radiation disease": new understanding and new solutions for a new disease in the era of cancer survivorship. Scand J Gastroenterol.

[10] Horch RE, Ludolph I, Cai A, Weber K, Grützmann R, Arkudas A (2020). Interdisciplinary surgical approaches in vaginal and perineal reconstruction of advanced rectal and anal female cancer patients. Front Oncol.

[11] Mirnezami A, Mirnezami R, Chandrakumaran K, Sasapu K, Sagar P, Finan P (2011). Increased local recurrence and reduced survival from colorectal cancer following anastomotic leak: systematic review and meta-analysis. Ann Surg.

